# Factors Associated with Suicidal Ideation Among Middle-Aged Korean Adults: A Cross-Sectional Analysis of the 2022–2023 Korea National Health and Nutrition Examination Survey

**DOI:** 10.3390/healthcare14203426

**Published:** 2026-10-14

**Authors:** Jung-Ha Son, Chul-Gyu Kim, Ji-Su Hong, Yun-Hee Oh, Ji-Yeon Choi

**Affiliations:** 1 College of Nursing, Chungbuk National University, Cheongju 28644, Republic of Korea; klecm92@chungbuk.ac.kr (J.-H.S.); unhezzang@cbnuh.or.kr (Y.-H.O.); chjy8888@cbnuh.or.kr (J.-Y.C.); 2 Department of Nursing, Chungbuk National University Hospital, Cheongju 28644, Republic of Korea; jisoo7751@chungbuk.ac.kr

**Keywords:** suicidal ideation, middle-aged, psychological stress, anxiety, residence characteristics, socioeconomic factors, health surveys, Republic of Korea

## Abstract

**Highlights:**

**What are the main findings?**

- High perceived stress (odds ratio (OR) = 8.54) and elevated anxiety symptoms (OR = 5.01) were the correlates most strongly associated with suicidal ideation among middle-aged Korean adults. Because depression was not adjusted for, these are total associations that may operate partly through depression.
- Socioeconomic disadvantage—lower educational attainment, economic inactivity, and low household income—remained independently associated with suicidal ideation after adjustment for household composition, health behaviors, and mental health.

**What are the implications of the main findings?**

- Perceived stress and elevated anxiety symptoms are candidate constructs for future suicide-risk screening research, but their predictive performance has yet to be established.
- Suicide prevention for middle-aged adults should extend beyond mental health services to include employment support and income security.

**Abstract:**

**Background/Objectives:** Suicide became the leading cause of death among Korean adults in their 40s in 2024. We identified sociodemographic, health behavior, and mental health correlates of suicidal ideation in mid-life without adjustment for depression, which overlaps with the outcome through Patient Health Questionnaire-9 item 9 and was measured in only one of the two survey years. **Methods:** This cross-sectional secondary analysis of the 9th Korea National Health and Nutrition Examination Survey (2022–2023) used complete data from 4715 of 5153 adults aged 40–64 years. Anxiety symptoms were assessed with the Generalized Anxiety Disorder-7 scale (screening threshold ≥ 10). Complex-sample logistic regression incorporated strata, clusters, and pooled weights, with all covariates prespecified. **Results:** Suicidal ideation prevalence was 3.8%. Independent correlates were high perceived stress (odds ratio (OR) = 8.54, 95% confidence interval (CI) 5.47–13.34), one-generation other-type household (OR = 6.81, 3.29–14.08; *n* = 59, 11 events), elevated anxiety symptoms (OR = 5.01, 3.02–8.32), middle school education or below (OR = 2.13, 1.12–4.03), economic inactivity (OR = 1.85, 1.25–2.73), poor perceived health (OR = 1.70, 1.13–2.55), and low household income (OR = 1.57, 1.08–2.28). Wave-specific adjustment for depression attenuated the stress and anxiety associations by 15–55% without eliminating them. **Conclusions:** Mid-life suicidal ideation was associated with mental health vulnerability, household composition, and socioeconomic disadvantage. The value of these cross-sectional associations for screening or risk stratification requires prospective evaluation.

## 1. Introduction

In 2024, suicide accounted for 26.0% of deaths among Korean adults in their 40s and, for the first time since national records began, became the leading cause of death in this age group; the age-specific suicide rate increased by 14.7% to 36.2 per 100,000 population, while suicide ranked second in causes of death among those in their 50s and fourth among those in their 60s [1]. Nationally, 14,872 suicide deaths were recorded, representing a 6.4% increase from the previous year, and the crude suicide rate of 29.1 per 100,000 was the highest since 2011 [2]. These trends indicate that suicide risk in Korea extends across the middle-aged population rather than being confined to a single age stratum, reflecting structural vulnerabilities and health inequities among adults aged 40–64 years. As life expectancy increases and mid-life occupies a larger proportion of the life course [3], the mental health burden during this period has growing consequences for individuals and society.

Middle-aged adults occupy a pivotal position in the life course, managing substantial occupational and social responsibilities while facing accumulating stressors [4]. Adults in their 40s often experience the convergence of child-rearing and parental care obligations, typifying the “sandwich generation,” in which caregiving demands and financial pressures are concentrated [5]. The chronic stress and emotional strain associated with these demands are recognized as important contributors to suicide vulnerability [6,7]. For adults in their 50s, household-level economic fragility, particularly fluctuations in spousal employment status and monthly income, may have a stronger influence on suicidal ideation than occupational instability alone [6]. Among adults in their 60s, physical aging coincides with the reorganization of social relationships following retirement [8], while accumulated loss experiences and psychosocial disruption may increase the likelihood that stress carried through mid-life manifests as suicidal behavior [5,9].

Although factors associated with suicidal ideation may differ across these decade-specific stages, mid-life suicidal ideation is often shaped not by discrete precipitating events but by persistent psychosocial stressors accumulated over the life course [6]. Depression is the most extensively studied predictor of suicidal ideation and has been consistently identified in prior research [6,10,11]. Beyond depression, stress perception, and anxiety disorders are associated with elevated suicide risk [12]. From a health behavior perspective, alcohol use is an important risk factor for suicide among middle-aged adults [7,13], and heavy alcohol consumption is associated with increased risks of both suicidal ideation and suicide attempts [14]. Smoking may also be an aggravating factor through its associations with nicotine dependence and mental health status [15]. Social-structural factors such as household composition are also relevant: middle-aged adults living in single-person households tend to report lower social support and greater social isolation, both of which are important pathways to suicidal ideation [15], whereas family support may be protective [7].

Existing studies on suicidal ideation among middle-aged Koreans have examined stress, depression, and social support [6]; single-person households [10]; and various subgroups within the 40–64-year range [11]. Further, several earlier analyses based on the Korea National Health and Nutrition Examination Survey (KNHANES) have addressed substantially overlapping middle-aged populations. The contribution of the present study is therefore incremental rather than foundational. It lies in three features: the use of the most recent (2022–2023) KNHANES wave, which postdates the 2024 rise in mid-life suicide mortality; the simultaneous consideration of household composition, socioeconomic position, perceived stress, and anxiety within a single prespecified model spanning the full 40–64-year range; and the explicit handling of depression as an analytic problem rather than as a routine covariate.

The last point requires explanation. Item 9 of the Patient Health Questionnaire-9 (PHQ-9) assesses passive suicidal ideation and therefore overlaps conceptually with our dependent variable [16]. Thus, entering the PHQ-9 total score as a predictor risks criterion contamination and may obscure the contributions of other, potentially modifiable risk factors; moreover, the PHQ-9 was administered in only one of the two survey years analyzed here. From a prevention perspective, identifying sociodemographic and health behavior correlates that are detectable before depression screening has practical value for community-based strategies.

This study used data from the 9th KNHANES to examine demographic, health behavior, and physical and mental health factors associated with suicidal ideation among adults aged 40–64 years. Specifically, we aimed to (1) identify differences in suicidal ideation according to demographic, health behavior, and physical and mental health characteristics, and (2) determine the factors associated with suicidal ideation in a prespecified multivariable model.

## 2. Materials and Methods

### 2.1. Design

This cross-sectional, descriptive correlational study was based on a secondary analysis of the 9th KNHANES (2022–2023). The study was reported in accordance with the STrengthening the Reporting of OBservational studies in Epidemiology (STROBE) statement for cross-sectional studies [17].

### 2.2. Participants

**Figure 1:**
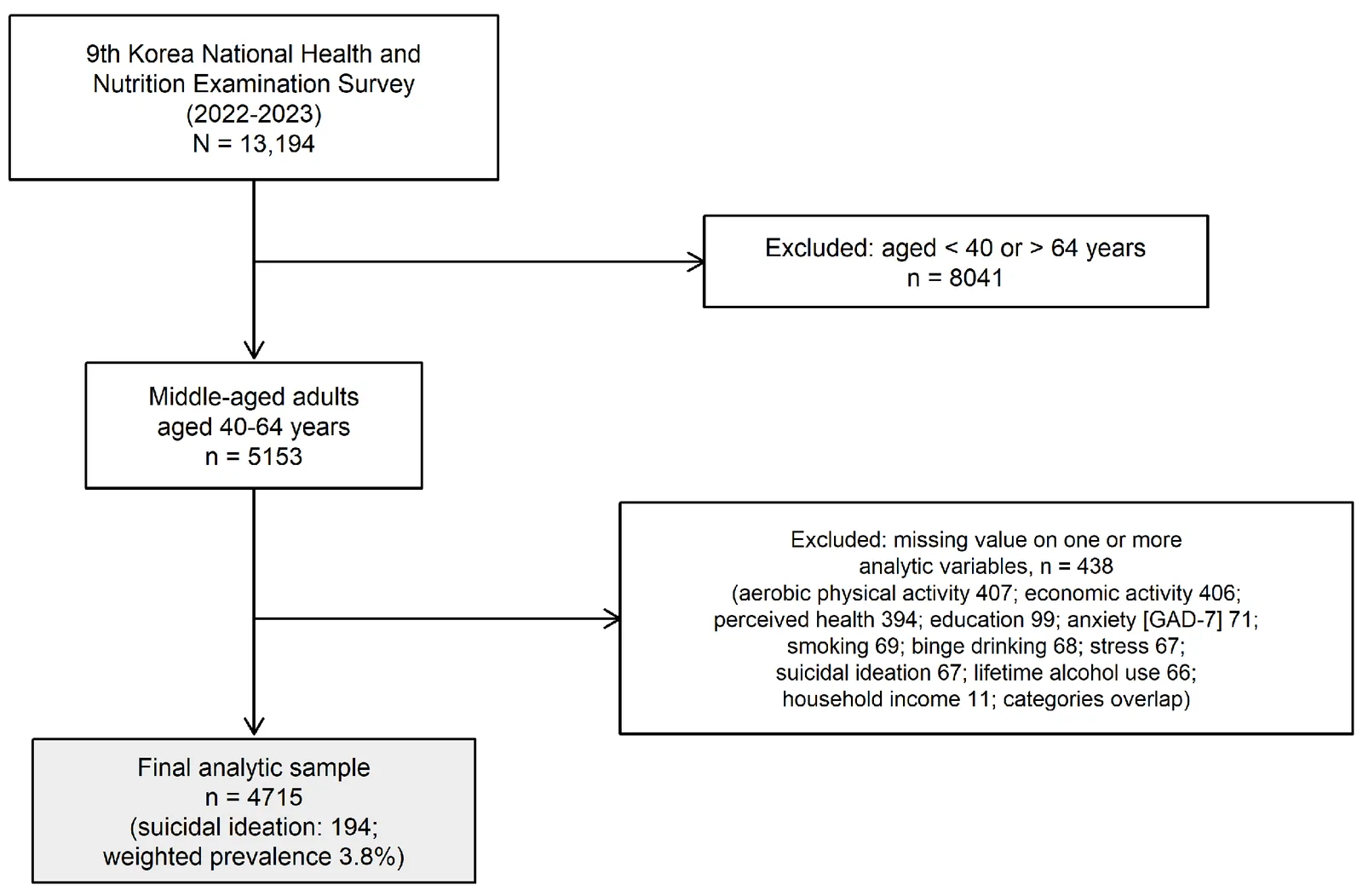
Flowchart of study participant selection. KNHANES, Korea National Health and Nutrition Examination Survey. Counts for individual variables sum to more than 438 because some participants had missing values on more than one variable.

The KNHANES is a nationally representative survey conducted annually by the Korea Disease Control and Prevention Agency (KDCA); its design and operation have been described in detail elsewhere [18]. Raw data from the 9th KNHANES (2022–2023) were released by the KDCA in January 2024. Of 13,194 total participants (6265 in 2022 and 6929 in 2023), 5153 were aged 40–64 years. A complete-case rule was applied across all analytic variables, and 438 individuals (8.5%) with a missing value on one or more of them were excluded, leaving 4715 participants for analysis (Figure 1). Missingness was concentrated in aerobic physical activity, economic activity, and perceived health status; variable-level counts are shown in Figure 1, and included and excluded participants are compared in Table S4, panel A.

### 2.3. Measures

#### 2.3.1. Sociodemographic Factors

Sociodemographic variables included sex, age group, household income, education level, living arrangement, marital status, and economic activity status. Age was grouped as 40–49, 50–59, and 60–64 years. Household income was dichotomized by quartile into low and lower-middle (lower two quartiles) versus upper-middle and high (upper two quartiles). Education level was classified as middle school or below, high school, or college or higher. Living arrangement was categorized into four types: one-generation single-person household, one-generation couple household, one-generation other-type household (e.g., living with siblings, relatives, or other non-spouse adults), and second- or higher-generation household. Marital status was classified as married or unmarried. Participants were considered economically active if they reported working for pay for 1 h or more per week or working 18 h or more per week as an unpaid family worker; all others were classified as economically inactive.

#### 2.3.2. Health-Related Factors

Health behavior variables included lifetime alcohol use, binge drinking frequency, current smoking status, and aerobic physical activity. Lifetime alcohol use was categorized as ever versus never having consumed at least one standard drink. Binge drinking frequency was classified as low (none or less than monthly) versus high (at least weekly), based on how often the participant consumed seven or more standard drinks (men) or five or more standard drinks (women) on a single occasion. Smoking status was categorized as current smoker versus non-current smoker (former smoker or lifetime non-smoker). Aerobic physical activity was classified as meeting guidelines if the participant engaged in moderate-intensity activity for at least 150 min per week, vigorous-intensity activity for at least 75 min per week, or an equivalent combination; all others were classified as not meeting guidelines.

Subjective health and mental health variables included perceived health status, stress perception, and anxiety symptoms. Stress perception was measured with a single item asking how much stress the participant usually feels in daily life and was dichotomized as high (feel very much or feel much) versus low (feel a little or hardly feel any); this is the cut point used for the KDCA’s published perceived-stress indicator in national health reporting, which permits direct comparison with prior Korean estimates. Perceived health status, recorded on five levels, was dichotomized as good (very good, good, or fair) versus poor (bad or very bad), as specified in the original analysis plan. Because the KDCA’s published perceived-health indicator divides the scale at a different point, the model was refitted under four codings of this variable (Table S4, panel C): the perceived-health estimate is sensitive to this choice, whereas every other estimate in the model is not.

Anxiety symptoms were assessed using the Generalized Anxiety Disorder-7 (GAD-7) scale, with a total score of 10 or higher classified as elevated anxiety symptoms and a score below 10 classified as no elevated anxiety symptoms [19]. The GAD-7 is a screening instrument; at this threshold it has been reported to yield approximately 89% sensitivity and 82% specificity against a structured diagnostic interview [19], but it does not establish a diagnosis under the Diagnostic and Statistical Manual of Mental Disorders, Fifth Edition (DSM-5), which requires clinical assessment of criteria not collected in the KNHANES. Participants above the threshold are therefore described throughout as having elevated anxiety symptoms, not generalized anxiety disorder.

#### 2.3.3. Suicidal Ideation

Suicidal ideation was assessed using a single item asking whether the participant had seriously thought about suicide during the past 12 months. Participants who answered “yes” were classified as having experienced suicidal ideation, whereas those who answered “no” were classified as not having experienced it.

### 2.4. Data Collection

The KNHANES assesses health behaviors, the prevalence of chronic diseases, and the dietary and nutritional status of the Korean population. Participants are recruited through stratified, multistage, probability-proportional cluster sampling based on administrative district and housing type, yielding a nationally representative, non-institutionalized civilian sample [18]. The present study used only publicly released, de-identified raw data files.

### 2.5. Data Analysis

Data were analyzed with SAS version 9.4 (SAS Institute Inc., Cary, NC, USA) and independently verified in R version 4.6.1 (R Core Team, Vienna, Austria) using the survey package, which reproduced every estimate. To account for the complex sample design, all analyses incorporated stratification variables (kstrata), clustering variables (psu), and sampling weights. The 9th KNHANES releases annual sampling weights (wt_itvex) constructed for single-year analysis; for the pooled two-year analysis, we applied the KDCA-recommended integrated weight for combining survey years, dividing the annual weight by the number of pooled years (wt_pooled = wt_itvex/2), and retained the original stratification and primary sampling unit variables without modification.

Frequencies and weighted proportions were calculated using complex sample frequency analysis. Group differences in the prevalence of suicidal ideation were tested using the Rao–Scott chi-square test. These univariate tests are reported for description only; they were not used to select variables for the multivariable model.

The multivariable model was prespecified on conceptual grounds, and every covariate was entered simultaneously and retained irrespective of its univariate statistical significance. Sex and age group were retained as demographic covariates by default. The remaining covariates were selected before analysis because each has prior evidential support as a correlate of suicidal ideation in middle-aged adults: socioeconomic position (household income, education level, economic activity status) [6,20,21,22,23]; social and household context (living arrangement, marital status) [10,15,24,25,26,27,28,29,30]; health behaviors (lifetime alcohol use, binge drinking frequency, current smoking, aerobic physical activity) [7,13,14,15,31,32,33]; and health and mental health status (perceived health status, elevated anxiety symptoms, stress perception) [6,11,12,20,34,35,36]. Therefore, the model contains 18 estimated parameters for 194 outcome events, approximately 11 events per parameter. Results are reported as odds ratios (ORs) with 95% confidence intervals (CIs). All tests were two-tailed, with statistical significance set at *p* < 0.05.

Depression was not entered into the principal model, for two reasons. Item 9 of the PHQ (Patient Health Questionnaire)-9 asks about thoughts of being better off dead or of self-harm and therefore overlaps conceptually with the dependent variable [16], so entering the PHQ-9 total score would risk criterion contamination; and the PHQ-9 was administered only in the 2022 wave of the 9th KNHANES, the 2023 wave instead carrying a single item on depressed mood lasting two weeks or longer, so no depression measure was common to both waves. The estimates are therefore total associations with suicidal ideation, which may include pathways operating through depression, rather than depression-adjusted direct effects.

Five sets of sensitivity analyses were conducted. Four were prespecified: the significance-based model used in the original analysis plan, refitted on the same sample for comparison with the prespecified model (Table S2); two wave-specific depression-adjusted models, entering a PHQ-8 score constructed from PHQ-9 items 1–8 with the suicidality item omitted, both dichotomously (≥10) and continuously, in the 2022 subsample, and the two-week depressed-mood item in the 2023 subsample (Table S3); a comparison of included and excluded age-eligible participants on all characteristics recorded for both groups (Table S4, panel A); and three assessments of the one-generation other-type household estimate, refitting the model with that category removed, by unweighted logistic regression with profile-likelihood CIs, and by collapsing the category with single-person households (Table S4, panel B). A fifth was added during revision: the model was refitted under four codings of perceived health status, reported alongside the level-specific prevalence (Table S4, panel C).

## 3. Results

### 3.1. Participants’ General Characteristics

Among the 4715 participants, 1934 (49.3%) were men and 2781 (50.7%) were women. The 50–59-year group was the largest (1856; 40.8%), followed by the 40–49-year group (1693; 38.6%) and 60–64-year group (1166; 20.6%). Regarding household income, 3227 (70.6%) participants were in the upper-middle or high category, and 1488 (29.4%) were in the low or lower-middle category. Regarding education, 2245 (49.5%) had completed college or higher, 1841 (39.0%) had completed high school, and 629 (11.5%) had completed middle school or below. Regarding living arrangement, 3030 (67.2%) lived in a second- or higher-generation household, 1055 (20.0%) in a one-generation couple household, 571 (11.5%) in a one-generation single-person household, and 59 (1.3%) in a one-generation other-type household. In total, 4338 (91.4%) were married, and 377 (8.6%) were unmarried; 3396 (74.3%) were economically active, and 1319 (25.7%) were economically inactive (Table 1). Included and excluded age-eligible participants did not differ in the prevalence of suicidal ideation (4.1% vs. 3.5%, *p* = 0.662), age, household income, education, living arrangement, or marital status, but excluded participants were more likely to be men, current smokers, and high-frequency binge drinkers (Table S4, panel A).

### 3.2. Health-Related Characteristics and Prevalence of Suicidal Ideation

Lifetime alcohol use was reported by 4380 participants (93.9%), whereas 335 (6.1%) reported no lifetime use. Binge drinking frequency was low for 3806 participants (78.2%) and high for 909 participants (21.8%). There were 826 current smokers (19.9%) and 3889 non-current smokers (80.1%). Perceived health status was good for 3910 participants (83.3%) and poor for 805 participants (16.7%). Stress perception was low for 3523 participants (74.6%) and high for 1192 participants (25.4%). Elevated anxiety symptoms were present among 184 participants (3.7%) and absent for 4531 participants (96.3%). Aerobic physical activity guidelines were met by 2200 participants (47.5%) and not met by 2515 participants (52.5%). Suicidal ideation during the past 12 months was reported by 194 participants (3.8%), whereas 4521 (96.2%) reported no suicidal ideation (Table 2).

### 3.3. Differences in Suicidal Ideation by General Characteristics

The prevalence of suicidal ideation did not differ significantly by sex or age group (*p* > 0.05). Significant differences were observed by household income, education level, living arrangement, marital status, and economic activity status (*p* < 0.05). Prevalence was higher in the low and lower–middle household income group (6.6%) than in the upper–middle and high group (2.6%) (χ^2^ = 41.80, *p* < 0.001) and was highest among participants with a middle school education or below (8.0%), followed by those with a high school education (4.1%) and those with a college education or higher (2.6%) (χ^2^ = 35.91, *p* < 0.001). Among living arrangement types, prevalence was highest in one-generation other-type households (23.1%), followed by single-person households (6.8%), couple households (3.5%), and second- or higher-generation households (3.0%) (χ^2^ = 82.10, *p* < 0.001). Prevalence was higher among unmarried participants (8.0%) than among married participants (3.4%) (χ^2^ = 21.22, *p* < 0.001) and among economically inactive participants (6.4%) than among economically active participants (2.9%) (χ^2^ = 30.46, *p* < 0.001) (Table 3).

### 3.4. Differences in Suicidal Ideation by Health-Related Characteristics

The prevalence of suicidal ideation did not differ significantly by lifetime alcohol use or aerobic physical activity (*p* > 0.05). Significant differences were observed for binge drinking frequency, smoking status, perceived health status, stress perception, and elevated anxiety symptoms (*p* < 0.05). Prevalence was higher among participants with high binge drinking frequency (5.3%) than among those with low frequency (3.4%) (χ^2^ = 8.40, *p* = 0.007) and among current smokers (6.0%) than among non-current smokers (3.2%) (χ^2^ = 15.55, *p* < 0.001). Participants with poor perceived health status had a substantially higher prevalence of suicidal ideation (10.6%) than those with good perceived health (2.4%) (χ^2^ = 119.69, *p* < 0.001). Prevalence was also markedly higher among participants with high stress perception (11.7%) than among those with low stress perception (1.1%) (χ^2^ = 276.04, *p* < 0.001). Among participants with elevated anxiety symptoms, prevalence was 31.2%, compared with 2.7% among those without them (χ^2^ = 373.32, *p* < 0.001) (Table 4).

### 3.5. Factors Associated with Suicidal Ideation

In the prespecified complex sample logistic regression model, household income, education level, living arrangement, economic activity status, perceived health status, elevated anxiety symptoms, and stress perception were significantly associated with suicidal ideation (Table 5). High stress perception was the strongest correlate (OR = 8.54, 95% CI 5.47–13.34, *p* < 0.001), followed by residence in a one-generation other-type household (OR = 6.81, 95% CI 3.29–14.08, *p* < 0.001) and elevated anxiety symptoms (OR = 5.01, 95% CI 3.02–8.32, *p* < 0.001). Middle school education or below (OR = 2.13, 95% CI 1.12–4.03, *p* = 0.020), economic inactivity (OR = 1.85, 95% CI 1.25–2.73, *p* = 0.002), poor perceived health status (OR = 1.70, 95% CI 1.13–2.55, *p* = 0.011), and low household income (OR = 1.57, 95% CI 1.08–2.28, *p* = 0.019) were also significantly associated with suicidal ideation.

Residence in a one-generation single-person household showed an elevated but imprecise association that did not reach statistical significance under prespecified adjustment (OR = 1.56, 95% CI 0.98–2.50, *p* = 0.062). Sex, age group, marital status, lifetime alcohol use, binge drinking frequency, current smoking, and aerobic physical activity were not independently associated with suicidal ideation once all covariates were entered simultaneously.

The one-generation other-type household category comprised 59 participants with 11 suicidal ideation events, and its estimate is correspondingly imprecise. Three robustness analyses supported an elevated association in this group while confirming the imprecision: the association persisted in an unweighted analysis with profile-likelihood CIs (OR = 4.85, 95% CI 1.88–11.71) and when the category was collapsed with single-person households into a one-generation non-couple household group (OR = 1.91, 95% CI 1.24–2.93, *p* = 0.003); removing the 59 participants altogether left all other estimates essentially unchanged (Table S4, panel B).

Refitting the significance-based specification used in the original analysis plan produced closely similar estimates for every covariate common to both models; the single-person household association was the only estimate whose statistical significance differed between the two specifications (Table S2).

### 3.6. Depression-Adjusted Sensitivity Analyses

As a depression measure common to both survey waves was unavailable, depression-adjusted models were fitted separately within each wave (Table S3). In the 2022 subsample (*n* = 2173, 92 events), the PHQ-8 constructed from items 1–8 correlated with the full PHQ-9 at *r* = 0.997. Adding the PHQ-8 to the prespecified model attenuated but did not eliminate the principal associations: high stress perception fell from OR = 7.73 (95% CI 3.87–15.44) to 6.59 (3.31–13.12) with a dichotomous PHQ-8 and to 5.48 (2.64–11.37) with a continuous PHQ-8, and elevated anxiety symptoms fell from 5.77 (2.61–12.75) to 4.02 (1.62–9.94) and 2.84 (1.10–7.37), respectively; all remained statistically significant. The one-generation other-type household association was largely unaffected (6.03 → 4.97 and 5.10), whereas the associations with poor perceived health and low household income were substantially attenuated.

In the 2023 subsample (*n* = 2542, 102 events), adding the two-week depressed-mood item produced a concordant pattern: high stress perception 9.09 (5.21–15.86) → 4.32 (2.22–8.42); elevated anxiety symptoms 4.96 (2.57–9.56) → 2.37 (1.19–4.71); one-generation other-type household 7.85 (2.69–22.91) → 7.02 (2.33–21.19); economic inactivity 2.11 (1.28–3.50) → 1.92 (1.06–3.50). Across both waves, and using two different depression instruments, adjustment for depression attenuated the stress and anxiety associations by approximately 15–55%, depending on the survey wave and on whether depression was modeled dichotomously or continuously, without eliminating them; this is consistent with the interpretation that the estimates in Table 5 represent total associations that partly operate through depression.

## 4. Discussion

This study examined the prevalence and correlates of suicidal ideation among Korean adults aged 40–64 years using nationally representative data. The prevalence was 3.8%, lower than the 5.4% reported by Hwang and Park [34] from the 6th KNHANES for the same age group, similar to the 3.6% reported by Lee and Choi [35] among Korean workers, and comparable to the 4.75% reported by Kim et al. [20] for the general adult population. This pattern is broadly consistent with the national decline in suicidal ideation reported by the Ministry of Health and Welfare, from 3.0% in 2018 to 2.1% in 2023 [37], and with the decline observed among people living in single-person households, from 14.6% in 2013 to 10.9% in 2021 [38]. However, prevalence in the present study was markedly higher in single-person households (6.8%) and one-generation other-type households (23.1%) than in the overall study population, while the suicide rate among adults in their 40s rose by 14.7% in 2024 [1]. Therefore, a comparatively low population prevalence of suicidal ideation should not be interpreted as indicating low overall suicide risk; early identification of individuals who report suicidal ideation and linkage to appropriate care remain public health priorities.

High stress perception was the strongest correlate of suicidal ideation (OR = 8.54), consistent with prior research focused on middle-aged adults [6,11,20,34]. The larger effect estimate observed here relative to that reported by Lee and Choi [35] is attributable in part to the fact that depression was not adjusted for in our principal model. Under the stress–diathesis model, suicidal behavior emerges from the interaction of environmental stress with biological vulnerability [39]; therefore, our estimate reflects both the association with stress and pathways involving depression. The wave-specific sensitivity analyses quantify the same: adjusting for the PHQ-8 in 2022 and for depressed mood in 2023 reduced the stress association by roughly 15% and 50% respectively, but it remained substantial and significant in both (Table S3). Cognitive-behavioral approaches and group psychoeducational programs targeting stress recognition may provide useful entry points for suicide prevention in this population [40]; workplace interventions incorporating stress education are also promising [41]. However, manager training alone has not consistently reduced employee psychological distress [42], indicating the need for multilevel approaches combining assessment, counselling, treatment, and linkage to welfare services [43].

Elevated anxiety symptoms were also strongly associated with suicidal ideation (OR = 5.01), consistent with prior studies focused on middle-aged adults [6,11,35]. Although the meta-analysis by Bentley et al. [12] identified anxiety as a statistically significant but relatively weak predictor of suicidal thoughts and behaviors, the 31.2% prevalence of suicidal ideation among participants above the GAD-7 screening threshold in this study is clinically noteworthy. At the same time, participants above that threshold accounted for fewer than one-third of all participants reporting suicidal ideation; thus, a GAD-7 score below 10 does not indicate low risk. The US Preventive Services Task Force recommends screening for anxiety disorders in adults aged 18–64 years [44], a range that encompasses the present population, and collaborative care in primary care settings reduces suicidal behavior [45]. However, we emphasize that these are cross-sectional associations in a general population sample, and that the GAD-7 threshold identifies elevated symptoms rather than a diagnosed anxiety disorder.

A distinction should be drawn between the risk factor associations reported here and clinical risk prediction. An OR of the magnitude observed for perceived stress or anxiety describes how the prevalence of suicidal ideation differs between groups; it does not establish that either measure can identify, in advance, which individuals will experience suicidal ideation. Demonstrating the same would require evidence on discrimination and calibration and a formal assessment of clinical utility using measures such as net benefit, none of which was evaluated here; current methodological guidance also cautions against framing such tools as a dichotomous stratification of patients into low- and high-risk categories, and favors estimated probabilities accompanied by impact assessment [46]. The performance that can realistically be expected is modest: in a recent evaluation of routine practice across 89,957 patients in 12 hospitals, clinicians’ structured suicide risk ratings predicted suicide attempt within 90 days with areas under the receiver operating characteristic curve of 0.60–0.77 depending on the care setting [47]. Nor does a cross-sectional design permit inference about temporal precedence, which any screening application would require. We therefore present perceived stress and elevated anxiety symptoms as candidate constructs for future screening research rather than as validated screening indicators. Further, we regard a sequential stress-to-GAD-7 risk stratification pathway as a hypothesis to be tested prospectively rather than a strategy supported by the present analysis.

The findings for living arrangement warrant particular attention and caution. Relative to second- or higher-generation households, the odds of suicidal ideation were highest among participants in one-generation other-type households (OR = 6.81), a category comprising 59 participants with 11 suicidal ideation events. The estimate is therefore imprecise, although its direction was confirmed in three robustness analyses (Table S4, panel B). The association for single-person households was smaller and did not reach statistical significance under prespecified adjustment (OR = 1.56, 95% CI 0.98–2.50), although it was significant in the significance-based specification and when covariates were added individually; we interpret it as a consistent but imprecise signal rather than an established independent correlate. What the two estimates jointly suggest is that prevention efforts focused solely on people living alone may overlook another group in which risk appears concentrated.

The distinction between what these variables measure and what they are taken to mean matters here. The KNHANES records household composition; it does not measure loneliness, perceived emotional support, or relationship quality, and we did not assess them. The mechanisms commonly invoked to explain such associations are therefore hypotheses drawn from external literature rather than findings of this study. Calati et al. [24] noted that suicidal thoughts and behaviors are associated not only with objective conditions such as living alone but also with subjective loneliness, and Chang et al. [25] reported that perceived loneliness and low social support are more strongly associated with suicide risk than the structural characteristics of social relationships. Social connection is in fact a multidimensional construct, encompassing structural features such as household composition, functional features such as perceived support, and the quality of relationships; these dimensions are conceptually distinct and are not interchangeable indicators [48]. The KNHANES records only the structural dimension. Evidence linking spousal absence to suicidal ideation in middle-aged adults [26,27], elevated risks of depression and suicidal ideation in single-person and solo-dwelling households [28,29], and a systematic review linking living alone to higher risks of suicide attempt and death [30] is likewise consistent with an emotional isolation pathway, but direct measurement of loneliness and perceived support would be required to establish one—including for the one-generation other-type households in which the risk observed here was concentrated.

Among health-related factors, poor perceived health status was independently associated with suicidal ideation (OR = 1.70), consistent with a previous Korean study focused on older adults [36]. The magnitude of this association depended on how the five-level measure was dichotomized (Table S4, panel C); therefore, we regard it as the least robust of the significant estimates. By contrast, current smoking and high binge drinking frequency were significant in univariate analyses but not in the prespecified multivariable model (OR = 1.52, 95% CI 0.94–2.46 and OR = 1.22, 95% CI 0.74–2.00, respectively). This pattern is consistent with smoking and heavy drinking acting as behavioral markers of underlying mental health vulnerability rather than as independent correlates of suicidal ideation, although a cross-sectional design cannot distinguish these interpretations. Kim et al. [31] reported that smoking was associated with suicidal ideation in conjunction with depression, and Kwon [32] similarly found smoking to be related to suicidal ideation in conjunction with stress, depression, and anxiety, while Harrison et al. [33] reported significant associations between smoking and both suicidal ideation and suicide attempts. Therefore, smoking cessation and alcohol reduction programs may benefit from integration with interventions that also address stress and anxiety.

Socioeconomic factors were also independently associated with suicidal ideation: economic inactivity (OR = 1.85), low household income (OR = 1.57), and middle school education or below (OR = 2.13). For middle-aged adults, economic participation extends beyond income to encompass social roles, self-esteem, and interpersonal networks; therefore, economic inactivity may be associated with greater psychological burden and social isolation. This interpretation is consistent with evidence that unemployment increases the risks of suicidal ideation, suicide attempts, and death [21] and that long-term unemployment is associated with elevated suicide risk [22]. The association between low income and suicidal ideation is likewise well-supported [6,20,23], and job and financial circumstances have been identified as important contributors to mid-life suicide [49]. Notably, the effect estimate for education exceeded that for household income and remained significant after adjustment for income, suggesting that educational attainment may capture cumulative life course vulnerability, including exposure to precarious working conditions and chronic stress, that is not fully reflected in current income. Minimum wage increases have been associated with reductions in suicide among adults with lower educational attainment [50], and government responses to economic downturns have been linked to attenuated increases in suicide [51]. Together, these findings support extending suicide prevention beyond the health sector to include employment support, income security, job retraining, and social welfare services. Notably, in the 2022 sensitivity analysis, the education association was unchanged by adjustment for depression, whereas the income and perceived health associations were substantially attenuated (Table S3), suggesting that educational attainment may index a form of cumulative life course vulnerability not mediated primarily by current depressive symptoms.

Taken together, these findings contribute recent, nationally representative evidence on mid-life suicidal ideation in Korea: they derive from the most recent (2022–2023) KNHANES wave, placing household composition, socioeconomic position, perceived stress, and elevated anxiety symptoms within a single prespecified model spanning the full 40–64-year range; further, the wave-specific analyses quantify how much of the stress and anxiety associations survives adjustment for depression.

### 4.1. Limitations

Several limitations should be considered when interpreting these findings. The cross-sectional design precludes inferences about temporal precedence or causality. Suicidal ideation was measured with a single item covering the past 12 months; such items have acceptable test–retest reliability and construct validity when evaluated against multi-item instruments [52], but they cannot grade severity, distinguish transient from persistent ideation, or capture progression to suicide planning or attempts, and a 12-month recall period is subject to recall bias. Perceived stress and perceived health status were likewise single items analyzed dichotomously, which discards information and may understate dose–response relationships; the perceived-health estimate in particular is attenuated to non-significance under the KDCA indicator cut point, although every other estimate in the model is essentially unchanged across codings (Table S4, panel C).

Depression was not adjusted for, partly to avoid criterion contamination and partly because the PHQ-9 was administered in the 2022 wave only (Section 2.5). The reported ORs are therefore total associations rather than depression-adjusted direct effects; the wave-specific analyses in Table S3 quantify the attenuation but cannot substitute for a pooled depression-adjusted model. Other potential confounders, including prior psychiatric history, unemployment duration, and financial debt, were unavailable, so residual confounding cannot be excluded. The analysis was also restricted to complete cases, excluding 438 of 5153 age-eligible participants (8.5%). Included and excluded participants did not differ in the prevalence of suicidal ideation, but did differ in sex and smoking status (Table S4, panel A); selection bias therefore cannot be excluded, and claims of representativeness should be read with this in mind.

Finally, the one-generation other-type household category contained only 59 participants with 11 suicidal ideation events. Although three robustness analyses supported the direction of this association, its magnitude should be regarded as provisional pending replication in larger samples. The sample was also restricted to community-dwelling adults, so the findings may not generalize to institutionalized populations.

### 4.2. Future Research Directions

Several priorities follow. Longitudinal designs are needed to establish temporal precedence between socioeconomic vulnerability, perceived stress, anxiety, depression, and suicidal ideation. Structured diagnostic assessment consistent with DSM-5 criteria would clarify whether the associations observed here for elevated anxiety symptoms extend to diagnosed anxiety disorders. Direct measurement of loneliness, perceived emotional support, and social network quality is required to test whether these constructs mediate the household composition associations, and the finding for one-generation other-type households should be replicated in larger samples capable of characterizing this heterogeneous category. Finally, any proposal to use perceived stress or GAD-7 scores for suicide-risk screening should be evaluated prospectively, with discrimination, calibration, and clinical utility reported at realistic base rates.

## 5. Conclusions

Using nationally representative data from the 9th KNHANES, this study found a 3.8% prevalence of suicidal ideation among Korean adults aged 40–64 years. In a prespecified multivariable model, the odds of suicidal ideation were significantly elevated among participants with low household income or educational attainment, those living in one-generation other-type households, economically inactive individuals, those reporting poor perceived health, and those with high perceived stress or elevated anxiety symptoms, the last two being the strongest correlates.

These findings indicate that mid-life suicidal ideation is associated with both socioeconomic vulnerability and mental health problems, and that suicide prevention for middle-aged adults should extend beyond individual psychological factors to integrate socioeconomic support with mental health services. The associations reported here identify where risk is concentrated in this population; they do not establish that any particular screening or risk stratification procedure would be effective. Whether a stepwise pathway based on perceived stress and the GAD-7 could identify at-risk middle-aged adults, and whether individuals in one-generation other-type and single-person households warrant specifically targeted intervention, require prospective evaluation in studies designed to assess predictive performance and intervention effectiveness.

## Figures and Tables

**Table 1:** Weighted Prevalence of Participants’ general characteristics (*N* = 4715).

Variables	Categories	*n*	%
Sex	Men	1934	49.3
	Women	2781	50.7
Age group (years)	40–49	1693	38.6
	50–59	1856	40.8
	60–64	1166	20.6
Household income	Low and lower-middle	1488	29.4
	Upper-middle and high	3227	70.6
Education level	Middle school or below	629	11.5
	High school	1841	39.0
	College or higher	2245	49.5
Living arrangement	One-generation, single-person	571	11.5
	One-generation, couple	1055	20.0
	One-generation, other	59	1.3
	Second or higher generation	3030	67.2
Marital status	Married	4338	91.4
	Unmarried	377	8.6
Economic activity	Active	3396	74.3
	Inactive	1319	25.7

Percentages are weighted estimates accounting for the complex sample design; unweighted counts are shown.

**Table 2:** Weighted Prevalence of Participants’ health-related characteristics (*N* = 4715).

Variables	Categories	*n*	%
Lifetime alcohol use	Yes	4380	93.9
	No	335	6.1
Binge drinking frequency	Low (none or <monthly)	3806	78.2
	High (≥weekly)	909	21.8
Smoking status	Non-current smoker	3889	80.1
	Current smoker	826	19.9
Perceived health status	Good	3910	83.3
	Poor	805	16.7
Stress perception	Low	3523	74.6
	High	1192	25.4
Elevated anxiety symptoms	No	4531	96.3
	Yes	184	3.7
Aerobic physical activity	Not meeting guidelines	2515	52.5
	Meeting guidelines	2200	47.5
Suicidal ideation	No	4521	96.2
	Yes	194	3.8

Percentages are weighted estimates accounting for the complex sample design; unweighted counts are shown. GAD-7, Generalized Anxiety Disorder-7. Elevated anxiety symptoms were defined as a GAD-7 total score of 10 or higher, a screening threshold that does not establish a clinical diagnosis.

**Table 3:** Differences in suicidal ideation by general characteristics (*N* = 4715).

Variables	Categories	Suicidal Ideation		*χ* ^2^	*p*
		Yes *n* (%)	No *n* (%)		
Sex	Men	66 (3.4)	1868 (96.6)	2.11	0.210
	Women	128 (4.2)	2653 (95.8)		
Age group (years)	40–49	61 (3.3)	1632 (96.7)	1.70	0.481
	50–59	79 (4.1)	1777 (95.9)		
	60–64	54 (4.0)	1112 (96.0)		
Household income	Low and lower-middle	102 (6.6)	1386 (93.4)	41.80	<0.001
	Upper-middle and high	92 (2.6)	3135 (97.4)		
Education level	Middle school or below	50 (8.0)	579 (92.0)	35.91	<0.001
	High school	79 (4.1)	1762 (95.9)		
	College or higher	65 (2.6)	2180 (97.4)		
Living arrangement	One-generation, single-person	42 (6.8)	529 (93.2)	82.10	<0.001
	One-generation, couple	39 (3.5)	1016 (96.5)		
	One-generation, other	11 (23.1)	48 (76.9)		
	Second or higher generation	102 (3.0)	2928 (97.0)		
Marital status	Married	166 (3.4)	4172 (96.6)	21.22	<0.001
	Unmarried	28 (8.0)	349 (92.0)		
Economic activity	Active	100 (2.9)	3296 (97.1)	30.46	<0.001
	Inactive	94 (6.4)	1225 (93.6)		

Percentages are weighted row estimates accounting for the complex sample design; unweighted counts are shown. χ^2^ denotes the Rao–Scott chi-square statistic.

**Table 4:** Differences in suicidal ideation by health-related characteristics (*N* = 4715).

Variables	Categories	Suicidal Ideation		*χ* ^2^	*p*
		Yes *n* (%)	No *n* (%)		
Lifetime alcohol use	Yes	179 (3.8)	4201 (96.2)	0.11	0.742
	No	15 (4.1)	320 (95.9)		
Binge drinking frequency	Low (none or <monthly)	145 (3.4)	3661 (96.6)	8.40	0.007
	High (≥weekly)	49 (5.3)	860 (94.7)		
Smoking status	Non-current smoker	140 (3.2)	3749 (96.8)	15.55	<0.001
	Current smoker	54 (6.0)	772 (94.0)		
Perceived health status	Good	106 (2.4)	3804 (97.6)	119.69	<0.001
	Poor	88 (10.6)	717 (89.4)		
Stress perception	Low	45 (1.1)	3478 (98.9)	276.04	<0.001
	High	149 (11.7)	1043 (88.3)		
Elevated anxiety symptoms	No	133 (2.7)	4398 (97.3)	373.32	<0.001
	Yes	61 (31.2)	123 (68.8)		
Aerobic physical activity	Not meeting guidelines	109 (3.8)	2406 (96.2)	0.01	0.926
	Meeting guidelines	85 (3.8)	2115 (96.2)		

Percentages are weighted row estimates accounting for the complex sample design; unweighted counts are shown. χ^2^ denotes the Rao–Scott chi-square statistic. GAD-7, Generalized Anxiety Disorder-7. Elevated anxiety symptoms were defined as a GAD-7 total score of 10 or higher, a screening threshold that does not establish a clinical diagnosis.

**Table 5:** Weighted Multivariate Logistic Regression Analysis of Factors associated with suicidal ideation (*N* = 4715).

Variables	Categories	OR (95% CI)	*p*
Sex	Men	1.00 (ref.)	
	Women	1.13 (0.71–1.80)	0.597
Age group (years)	40–49	1.00 (ref.)	
	50–59	1.22 (0.80–1.86)	0.351
	60–64	1.26 (0.73–2.15)	0.402
Household income	Upper-middle and high	1.00 (ref.)	
	Low and lower-middle	1.57 (1.08–2.28)	0.019
Education level	College or higher	1.00 (ref.)	
	High school	1.26 (0.83–1.91)	0.270
	Middle school or below	2.13 (1.12–4.03)	0.020
Living arrangement	Second or higher generation	1.00 (ref.)	
	One-generation, single-person	1.56 (0.98–2.50)	0.062
	One-generation, couple	1.09 (0.64–1.84)	0.760
	One-generation, other	6.81 (3.29–14.08)	<0.001
Marital status	Married	1.00 (ref.)	
	Unmarried	1.19 (0.62–2.29)	0.600
Economic activity	Active	1.00 (ref.)	
	Inactive	1.85 (1.25–2.73)	0.002
Lifetime alcohol use	Yes	1.00 (ref.)	
	No	0.88 (0.39–1.98)	0.761
Binge drinking frequency	Low (none or <monthly)	1.00 (ref.)	
	High (≥weekly)	1.22 (0.74–2.00)	0.440
Smoking status	Non-current smoker	1.00 (ref.)	
	Current smoker	1.52 (0.94–2.46)	0.086
Aerobic physical activity	Meeting guidelines	1.00 (ref.)	
	Not meeting guidelines	0.84 (0.58–1.23)	0.375
Perceived health status	Good	1.00 (ref.)	
	Poor	1.70 (1.13–2.55)	0.011
Elevated anxiety symptoms	No	1.00 (ref.)	
	Yes	5.01 (3.02–8.32)	<0.001
Stress perception	Low	1.00 (ref.)	
	High	8.54 (5.47–13.34)	<0.001

OR, odds ratio; CI, confidence interval; ref., reference category. Complex sample multiple logistic regression with strata, cluster, and pooled sampling weights applied. All covariates shown were prespecified on conceptual grounds, entered simultaneously, and retained irrespective of univariate significance; the model contains 18 estimated parameters for 194 outcome events. Depression was not adjusted for, so these are total associations ( Section 2.5; Table S3). Elevated anxiety symptoms were defined as a GAD-7 total score of 10 or higher.

## Data Availability

The data analyzed in this study are publicly available from the Korea National Health and Nutrition Examination Survey (KNHANES) database maintained by the Korea Disease Control and Prevention Agency at https://knhanes.kdca.go.kr (accessed on 28 August 2026). Access requires free registration as a data user and submission of the standard data use agreement, after which the de-identified raw data files are downloadable without charge or further restriction; no additional institutional review board approval is required from the KDCA for secondary analysis of the released files. The complete analytic code used in this study—an R script that reproduces every reported estimate, all variable derivation rules, and full specifications of the principal and sensitivity models—is provided as Supplementary File S2, so that all reported results can be reproduced exactly from the publicly available data.

## Supplementary Materials

The following supporting information can be downloaded at: https://www.mdpi.com/article/10.3390/healthcare14203426/s1, Table S1: STROBE Statement: checklist of items that should be included in reports of cross-sectional studies; Table S2: Comparison of the prespecified and significance-based multivariable models; Table S3: Depression-adjusted sensitivity analyses; Table S4: Robustness analyses; File S2: Analytic code, variable derivation rules, and model specifications.

## Abbreviations

The following abbreviations are used in this manuscript:

CI	Confidence interval
GAD-7	Generalized Anxiety Disorder-7 scale
KDCA	Korea Disease Control and Prevention Agency
KNHANES	Korea National Health and Nutrition Examination Survey
OR	Odds ratio
PHQ-9	Patient Health Questionnaire-9
STROBE	Strengthening the Reporting of Observational Studies in Epidemiology
