# Supplementary material for: Factors Associated with Suicidal Ideation Among Middle-Aged Korean Adults: A Cross-Sectional Analysis of the 2022–2023 Korea National Health and Nutrition Examination Survey
Source: Healthcare (Basel). 2026 Oct 14;14(20):3426. doi: 10.3390/healthcare14203426 (PMC13704684; doi:10.3390/healthcare14203426)
Supplement: Supplementary file 1 [file healthcare-14-03426-s001.zip › healthcare-4578331-supplementary File S2.pdf]

File S2. Analytic code, variable derivation rules, and model specifications

Manuscript: healthcare-4578331 — *Factors Associated with Suicidal Ideation among Middle-Aged Korean Adults: A Cross-Sectional Analysis of the 2022-2023 Korea National Health and Nutrition Examination Survey*

This file contains the complete analytic code for the study. Running it against the two publicly available 9th KNHANES raw data files (`hn22_all.sas7bdat` and `hn23_all.sas7bdat`, obtainable from https://knhanes.kdca.go.kr) reproduces every value reported in Tables 1-5 and in Supplementary Tables S2-S4.

The script is organised as follows:

Section	Contents
1	Loading and pooling the two survey waves, and construction of the integrated two-year sampling weight
2	Variable derivation rules for every analytic variable, including the treatment of missing, “don’t know” and refused responses
3	Definition of the analytic sample (complete-case rule applied across all analytic variables)
4	Complex survey design object and reference categories
5	Tables 1-4: distributions and univariate differences
6	Table 5: the principal prespecified multivariable model
7	Table S2: comparison with the significance-based specification
8	Table S3: wave-specific depression-adjusted sensitivity analyses
9	Table S4, panel A: comparison of included and excluded participants
10	Table S4, panel B: robustness of the one-generation other-type household estimate

The published analysis was performed in SAS version 9.4 using PROC SURVEYFREQ and PROC SURVEYLOGISTIC. The R implementation below, which uses the survey package, was written as an independent verification of that analysis and reproduces all reported estimates.

```
###########################################################################
#########
## Supplementary File S2 – Analytic code, variable derivation rules and
 model
## specifications
##
## Manuscript: Factors Associated with Suicidal Ideation among Middle-A
ged
##             Korean Adults: A Cross-Sectional Analysis of the 2022-20
23 Korea
##             National Health and Nutrition Examination Survey
##
## Data source: 9th KNHANES (2022, 2023), Korea Disease Control and Pre
vention
##              Agency, https://knhanes.kdca.go.kr
##              Required raw files: hn22_all.sas7bdat, hn23_all.sas7bda
t
##
## This script reproduces every number reported in Tables 1-5 and
## Supplementary Tables S2-S4. The published analysis was performed in
## SAS 9.4 (PROC SURVEYFREQ / PROC SURVEYLOGISTIC); this R implementati
on
## reproduces all estimates and is provided for independent verificatio
n.
###########################################################################
#########

suppressMessages({
  library(haven)    # read_sas
  library(dplyr)
  library(survey)
})
options(survey.lonely.psu = "adjust")

## Set to the directory holding hn22_all.sas7bdat and hn23_all.sas7bdat
DATA_DIR <- "."

###########################################################################
#########
## 1. Load and pool the two survey waves
###########################################################################
#########
```

```
keepv <- c("year", "kstrata", "psu", "wt_itvex", "sex", "age", "ho_incm
", "edu",
           "genertn", "marri_1", "EC1_1", "BD1", "BD2_31", "BS3_1",
           paste0("BE3_", c(71:78, 81:88, 91:94)),
           "D_1_1", "BP1", "BP5", "mh_GAD_S", "BP6_10",
           paste0("BP_PHQ_", 1:9))

read_keep <- function(f) {
  d <- read_sas(file.path(DATA_DIR, f))
  d[, intersect(keepv, names(d)), drop = FALSE]
}
d <- bind_rows(read_keep("hn22_all.sas7bdat"), read_keep("hn23_all.sas7
bdat"))

## Integrated two-year weight (KDCA guidance for pooling survey years):
## divide the single-year interview+examination weight by the number of
 years.
d$wt_pooled <- d$wt_itvex / 2
d$age4064   <- as.integer(d$age >= 40 & d$age <= 64)

#######################################################################
#########
## 2. Variable derivation
##
## NOTE ON MISSING VALUES. Every derivation below returns NA for a miss
ing,
## "don't know" or "refused" response. Values are never folded into a
## substantive category. The complete-case rule in section 3 is then ap
plied
## across ALL analytic variables, not only those in the final model.
#######################################################################
#########

## Outcome: serious suicidal ideation in the past 12 months (BP6_10: 1=
yes, 2=no)
d$SI <- with(d, ifelse(BP6_10 == 1, 1L, ifelse(BP6_10 == 2, 0L, NA_inte
ger_)))

## --- Sociodemographic ---------------------------------------------
--------
d$sex_1     <- ifelse(d$sex == 1, 0L, 1L)                  # 0 = men, 1
 = women
d$age_group <- with(d, ifelse(age <= 49, 1L, ifelse(age <= 59, 2L, 3L)))

## Household income quartile: 0 = low / lower-middle, 1 = upper-middle
/ high
d$inc_2 <- ifelse(is.na(d$ho_incm), NA_integer_, as.integer(d$ho_incm >
 2))
```

```
## Education: 0 = middle school or below, 1 = high school, 2 = college
or higher
d$edu_3 <- with(d, ifelse(is.na(edu), NA_integer_,
                   ifelse(edu <= 2, 0L, ifelse(edu == 3, 1L, 2L))))

## Living arrangement (genertn): 0 = one-generation single-person,
## 1 = one-generation couple, 2 = one-generation other,
## 3 = second or higher generation
d$fam_4 <- with(d, ifelse(genertn == 1, 0L,
                   ifelse(genertn == 2, 1L,
                   ifelse(genertn == 3, 2L,
                   ifelse(genertn >= 4 & genertn <= 7, 3L, NA_integer
_)))))

## Marital status: 1 = married, 0 = unmarried
d$mari_2 <- with(d, ifelse(marri_1 == 1, 1L, ifelse(marri_1 == 2, 0L, N
A_integer_)))

## Economic activity: 1 = active, 0 = inactive
d$ec_2 <- with(d, ifelse(EC1_1 == 1, 1L, ifelse(EC1_1 == 2, 0L, NA_inte
ger_)))

## --- Health behaviour -----------------------------------------------
--------
## Lifetime alcohol use (BD1: 1 = never, 2 = ever): 1 = yes, 0 = no
d$alc_ever <- with(d, ifelse(BD1 == 2, 1L, ifelse(BD1 == 1, 0L, NA_inte
ger_)))

## Binge drinking frequency (BD2_31): 1 = high (>= weekly), 0 = low
## codes 1-3 = less than weekly, 4-5 = weekly or more, 8 = no drinking
in past
## year (treated as low), 9 = don't know / refused (treated as missing)
d$binge_2 <- with(d, ifelse(BD2_31 %in% c(1, 2, 3, 8), 0L,
                     ifelse(BD2_31 %in% c(4, 5), 1L, NA_integer_)))

## Current smoking (BS3_1): 1 = current smoker, 0 = non-current smoker
d$smk_2 <- with(d, ifelse(BS3_1 %in% c(1, 2), 1L,
                   ifelse(BS3_1 %in% c(3, 8), 0L, NA_integer_)))

## Aerobic physical activity: 1 = meeting guidelines, 0 = not meeting
valid_min <- function(x) !(is.na(x) | x %in% c(88, 99))
segment <- function(yes, days, hh, mm) {
  ok <- (yes == 1) & (days %in% 1:7) & valid_min(hh) & valid_min(mm)
  ok[is.na(ok)] <- FALSE
  ifelse(ok, days * (hh * 60 + mm), NA_real_)
}
rsum <- function(M) apply(M, 1, function(v) if (all(is.na(v))) NA_real_
 else sum(v, na.rm = TRUE))
```

```r
vig_work <- with(d, segment(BE3_71, BE3_72, BE3_73, BE3_74))
mod_work <- with(d, segment(BE3_81, BE3_82, BE3_83, BE3_84))
vig_leis <- with(d, segment(BE3_75, BE3_76, BE3_77, BE3_78))
mod_leis <- with(d, segment(BE3_85, BE3_86, BE3_87, BE3_88))
walk     <- with(d, segment(BE3_91, BE3_92, BE3_93, BE3_94))

vig_total <- rsum(cbind(vig_work, vig_leis))
mod_total <- rsum(cbind(mod_work, walk, mod_leis))
combined  <- ifelse(is.na(vig_total * 2) & is.na(mod_total), NA_real_,
                    rsum(cbind(vig_total * 2, mod_total)))

pa_valid <- with(d,
  ((BE3_71 == 1 & BE3_72 %in% 1:7 & !is.na(vig_work)) | BE3_71 == 2) &
  ((BE3_81 == 1 & BE3_82 %in% 1:7 & !is.na(mod_work)) | BE3_81 == 2) &
  ((BE3_75 == 1 & BE3_76 %in% 1:7 & !is.na(vig_leis)) | BE3_75 == 2) &
  ((BE3_85 == 1 & BE3_86 %in% 1:7 & !is.na(mod_leis)) | BE3_85 == 2) &
  ((BE3_91 == 1 & BE3_92 %in% 1:7 & !is.na(walk))     | BE3_91 == 2))
pa_valid[is.na(pa_valid)] <- FALSE

d$pa_2 <- ifelse(pa_valid,
                 as.integer((mod_total >= 150 & !is.na(mod_total)) |
                            (vig_total >=  75 & !is.na(vig_total)) |
                            (combined  >= 150 & !is.na(combined))),
                 NA_integer_)

## --- Subjective health and mental health ----------------------------
--------
## Perceived health (D_1_1: 1 very good ... 5 very bad): 1 = good, 0 =
poor
d$health_2 <- with(d, ifelse(D_1_1 %in% 1:3, 1L, ifelse(D_1_1 %in% 4:5,
 0L, NA_integer_)))

## Perceived stress (BP1: 1 very much ... 4 hardly any): 1 = high, 0 =
low
d$stress_2 <- with(d, ifelse(BP1 %in% c(1, 2), 1L, ifelse(BP1 %in% c(3,
 4), 0L, NA_integer_)))

## Elevated anxiety symptoms: GAD-7 total >= 10 (screening threshold)
d$gad_2 <- with(d, ifelse(is.na(mh_GAD_S), NA_integer_, as.integer(mh_G
AD_S >= 10)))

## --- Depression measures (sensitivity analyses only) ----------------
--------
## 2022 wave only: PHQ-8 = sum of PHQ-9 items 1-8, omitting the suicida
lity item
phq_items <- as.matrix(d[, paste0("BP_PHQ_", 1:9)])
phq_ok    <- apply(phq_items, 1, function(x) all(!is.na(x) & x %in% 0:
3))
```

```
d$phq8    <- ifelse(phq_ok, rowSums(phq_items[, 1:8]), NA_real_)
d$phq9    <- ifelse(phq_ok, rowSums(phq_items),        NA_real_)
d$phq8_hi <- ifelse(is.na(d$phq8), NA_integer_, as.integer(d$phq8 >= 1
0))

## 2023 wave only: single item, depressed mood lasting >= 2 weeks in pa
st year
d$depmood <- with(d, ifelse(BP5 == 1, 1L, ifelse(BP5 == 2, 0L, NA_integ
er_)))

########################################################################
#########
## 3. Analytic sample: age 40-64 with complete data on all analytic var
iables
########################################################################
#########

analytic_vars <- c("SI", "inc_2", "edu_3", "fam_4", "mari_2", "ec_2",
                   "alc_ever", "binge_2", "smk_2", "pa_2",
                   "health_2", "stress_2", "gad_2")

d$analytic <- as.integer(d$age4064 == 1 & complete.cases(d[, analytic_v
ars]))

cat("Age-eligible (40-64):", sum(d$age4064 == 1), "\n")            # 51
53
cat("Excluded (incomplete):", sum(d$age4064 == 1) - sum(d$analytic), "\
n")  # 438
cat("Analytic sample:", sum(d$analytic), "\n")                      # 4
715
cat("Suicidal ideation events:", sum(d$SI[d$analytic == 1] == 1), "\n")
     # 194

########################################################################
#########
## 4. Complex survey design
########################################################################
#########

design   <- svydesign(ids = ~psu, strata = ~kstrata, weights = ~wt_pool
ed,
                      data = d, nest = TRUE)

## Reference categories, applied to any subset of the design
set_refs <- function(dsn) update(dsn,
  sex_1     = relevel(factor(sex_1),     ref = "0"),   # men
  age_group = relevel(factor(age_group), ref = "1"),   # 40-49
  inc_2     = relevel(factor(inc_2),     ref = "1"),   # upper-middle /
 high
```

```
  edu_3     = relevel(factor(edu_3),     ref = "2"),   # college or hig
her
  fam_4     = relevel(factor(fam_4),     ref = "3"),   # second or high
er generation
  mari_2    = relevel(factor(mari_2),    ref = "1"),   # married
  ec_2      = relevel(factor(ec_2),      ref = "1"),   # economically a
ctive
  alc_ever  = relevel(factor(alc_ever),  ref = "1"),   # lifetime alcoh
ol use: yes
  binge_2   = relevel(factor(binge_2),   ref = "0"),   # low binge freq
uency
  smk_2     = relevel(factor(smk_2),     ref = "0"),   # non-current sm
oker
  pa_2      = relevel(factor(pa_2),      ref = "1"),   # meeting guidel
ines
  health_2  = relevel(factor(health_2),  ref = "1"),   # good perceived
 health
  gad_2     = relevel(factor(gad_2),     ref = "0"),   # no elevated an
xiety symptoms
  stress_2  = relevel(factor(stress_2),  ref = "0"))   # low perceived
stress

dz <- set_refs(subset(design, analytic == 1))

report <- function(fit, label) {
  est <- exp(cbind(coef(fit), confint(fit)))
  p   <- summary(fit)$coefficients[, 4]
  out <- data.frame(
    term = rownames(est),
    OR   = sprintf("%.2f (%.2f-%.2f)", est[, 1], est[, 2], est[, 3]),
    p    = ifelse(p < 0.001, "<0.001", sprintf("%.3f", p)),
    stringsAsFactors = FALSE)
  cat("\n===", label, "  n =", nobs(fit), "===\n")
  print(out[-1, ], row.names = FALSE)
  invisible(out)
}

########################################################################
#########
## 5. Tables 1-4: distributions and univariate differences
########################################################################
#########

tab_vars <- c("sex_1", "age_group", "inc_2", "edu_3", "fam_4", "mari_2",
 "ec_2",
              "alc_ever", "binge_2", "smk_2", "pa_2", "health_2", "stre
ss_2",
              "gad_2", "SI")
```

```r
cat("\n---- Tables 1 and 2: unweighted n and weighted % ----\n")
a <- subset(d, analytic == 1)
for (v in tab_vars) {
  pct <- svymean(as.formula(paste0("~factor(", v, ")")), dz) * 100
  cnt <- table(a[[v]])
  cat("\n", v, "\n", sep = "")
  for (k in names(cnt))
    cat(sprintf("   %-4s %5d %6.1f\n", k, cnt[[k]], pct[[paste0("factor
(", v, ")", k)]]))
}

cat("\n---- Tables 3 and 4: suicidal ideation by group, Rao-Scott chi-s
quare ----\n")
for (v in setdiff(tab_vars, "SI")) {
  m  <- svyby(~SI, as.formula(paste0("~", v)), dz, svymean, na.rm = TRU
E)
  rs <- svychisq(as.formula(paste0("~SI+", v)), dz, statistic = "Chisq")
  cat("\n", v, "\n", sep = "")
  for (i in seq_len(nrow(m))) {
    k <- as.numeric(as.character(m[[1]][i]))
    cat(sprintf("   %-4s yes %3d (%4.1f%%)  no %4d (%4.1f%%)\n", m[[1]]
[i],
                sum(a$SI[a[[v]] == k] == 1), m$SI[i] * 100,
                sum(a$SI[a[[v]] == k] == 0), (1 - m$SI[i]) * 100))
  }
  cat(sprintf("   Rao-Scott chi2 = %.2f, p = %s\n", rs$statistic,
              ifelse(rs$p.value < 0.001, "<0.001", sprintf("%.3f", rs$p.
value))))
}

#######################################################################
#########
## 6. Table 5: principal prespecified multivariable model
##
## All covariates are entered simultaneously and retained irrespective
of
## univariate statistical significance. No variable is selected or remo
ved on
## the basis of a univariate test.
#######################################################################
#########

PRESPECIFIED <- SI ~ sex_1 + age_group + inc_2 + edu_3 + fam_4 + mari_2
 + ec_2 +
                     alc_ever + binge_2 + smk_2 + pa_2 + health_2 + gad
_2 + stress_2

m_main <- svyglm(PRESPECIFIED, design = dz, family = quasibinomial())
report(m_main, "TABLE 5  prespecified multivariable model")
```

```
#########################################################################
#########
## 7. Table S2: comparison with the significance-based specification
#########################################################################
#########

SIGNIFICANCE_BASED <- SI ~ inc_2 + edu_3 + fam_4 + ec_2 + smk_2 + healt
h_2 +
                           gad_2 + stress_2

report(svyglm(SIGNIFICANCE_BASED, design = dz, family = quasibinomial
()),
       "TABLE S2  significance-based model (comparison only)")

#########################################################################
#########
## 8. Table S3: depression-adjusted sensitivity analyses
##
## The PHQ-9 was administered in the 2022 wave only; the 2023 wave carr
ies a
## single two-week depressed-mood item. Depression-adjusted models are
## therefore fitted separately within each wave.
#########################################################################
#########

## -- Panel A: 2022 wave, PHQ-8 (items 1-8, suicidality item omitted) -
--------
z22 <- set_refs(subset(design, analytic == 1 & year == 2022 & !is.na(ph
q8)))
z22 <- update(z22, phq8_hi = relevel(factor(phq8_hi), ref = "0"))

a22 <- subset(d, analytic == 1 & year == 2022 & !is.na(phq8))
cat("\n2022 subsample n =", nrow(a22), " events =", sum(a22$SI == 1),
    " PHQ-8 >= 10:", sum(a22$phq8 >= 10),
    " cor(PHQ-8, PHQ-9) =", round(cor(a22$phq8, a22$phq9), 3), "\n")

report(svyglm(PRESPECIFIED, design = z22, family = quasibinomial()),
       "TABLE S3A-1  2022, without depression")
report(svyglm(update(PRESPECIFIED, . ~ . + phq8_hi), design = z22, fami
ly = quasibinomial()),
       "TABLE S3A-2  2022, + PHQ-8 >= 10")
report(svyglm(update(PRESPECIFIED, . ~ . + phq8), design = z22, family
= quasibinomial()),
       "TABLE S3A-3  2022, + PHQ-8 continuous")

## -- Panel B: 2023 wave, two-week depressed mood ---------------------
--------
z23 <- set_refs(subset(design, analytic == 1 & year == 2023 & !is.na(de
```

```
pmood)))
z23 <- update(z23, depmood = relevel(factor(depmood), ref = "0"))

a23 <- subset(d, analytic == 1 & year == 2023 & !is.na(depmood))
cat("\n2023 subsample n =", nrow(a23), " events =", sum(a23$SI == 1),
    " depressed mood:", sum(a23$depmood == 1), "\n")

report(svyglm(PRESPECIFIED, design = z23, family = quasibinomial()),
       "TABLE S3B-1  2023, without depression")
report(svyglm(update(PRESPECIFIED, . ~ . + depmood), design = z23, fami
ly = quasibinomial()),
       "TABLE S3B-2  2023, + two-week depressed mood")

########################################################################
#########
## 9. Table S4 panel A: included versus excluded age-eligible participa
nts
########################################################################
#########

e <- subset(d, age4064 == 1)
e$included <- e$analytic == 1

cat("\n---- TABLE S4A  included (n =", sum(e$included), ") vs excluded
(n =",
    sum(!e$included), ") ----\n")
for (v in c("sex_1", "age_group", "inc_2", "edu_3", "fam_4", "mari_2",
            "binge_2", "smk_2", "SI")) {
  tb <- table(e$included, e[[v]])
  pc <- round(100 * prop.table(tb, 1), 1)
  pv <- suppressWarnings(chisq.test(tb)$p.value)
  cat(sprintf("%-10s incl: %-28s excl: %-28s p = %s\n", v,
      paste(sprintf("%s=%.1f%%", colnames(tb), pc["TRUE", ]),  collapse
 = " "),
      paste(sprintf("%s=%.1f%%", colnames(tb), pc["FALSE", ]), collapse
 = " "),
      ifelse(pv < 0.001, "<0.001", sprintf("%.3f", pv))))
}
cat(sprintf("age  incl mean %.1f, excl mean %.1f, p = %.3f\n",
    mean(e$age[e$included]), mean(e$age[!e$included]),
    t.test(age ~ included, data = e)$p.value))

########################################################################
#########
## 10. Table S4 panel B: robustness of the one-generation other-type es
timate
########################################################################
#########
```

```
## (i) refit with the category removed
report(svyglm(PRESPECIFIED, design = set_refs(subset(design, analytic =
= 1 & fam_4 != 2)),
              family = quasibinomial()),
       "TABLE S4B-i  excluding one-generation other-type households")

## (ii) unweighted logistic regression with profile-likelihood CIs
a$fam <- relevel(factor(a$fam_4), ref = "3")
m_unw <- glm(SI ~ factor(sex_1) + factor(age_group) + factor(inc_2) + f
actor(edu_3) +
                  fam + factor(mari_2) + factor(ec_2) + factor(alc_ever)
 +
                  factor(binge_2) + factor(smk_2) + factor(pa_2) +
                  factor(health_2) + factor(gad_2) + factor(stress_2),
             data = a, family = binomial)
ci_unw <- suppressMessages(confint(m_unw))
cat("\nTABLE S4B-ii  unweighted, profile-likelihood CIs\n")
for (j in grep("^fam", names(coef(m_unw))))
  cat(sprintf("   %-8s OR = %.2f (%.2f-%.2f)\n", names(coef(m_unw))[j],
              exp(coef(m_unw)[j]), exp(ci_unw[j, 1]), exp(ci_unw[j,
2])))

## (iii) collapse one-generation other-type with single-person househol
ds
d$fam_collapsed <- ifelse(d$fam_4 %in% c(0, 2), 0L, ifelse(d$fam_4 == 1,
 1L, 3L))
design2 <- svydesign(ids = ~psu, strata = ~kstrata, weights = ~wt_poole
d,
                     data = d, nest = TRUE)
zc <- set_refs(subset(design2, analytic == 1))
zc <- update(zc, fam_collapsed = relevel(factor(fam_collapsed), ref = "
3"))
report(svyglm(update(PRESPECIFIED, . ~ . - fam_4 + fam_collapsed),
              design = zc, family = quasibinomial()),
       "TABLE S4B-iii  one-generation non-couple household (collapsed)")

#######################################################################
#########
## End of file
#######################################################################
#########
```
