# Supplementary material for: Factors Associated with Suicidal Ideation Among Middle-Aged Korean Adults: A Cross-Sectional Analysis of the 2022–2023 Korea National Health and Nutrition Examination Survey
Source: Healthcare (Basel). 2026 Oct 14;14(20):3426. doi: 10.3390/healthcare14203426 (PMC13704684; doi:10.3390/healthcare14203426)
Supplement: Supplementary file 1 [file healthcare-14-03426-s001.zip › healthcare-4578331-supplementary.pdf]

Supplementary Materials

Manuscript: Factors Associated with Suicidal Ideation among Middle-Aged Korean Adults: A Cross-Sectional Analysis of the 2022–2023 Korea National Health and Nutrition Examination Survey (healthcare-4578331, revised version).

Table S1. STROBE Statement: checklist of items that should be included in reports of cross-sectional studies

Locations are given by manuscript section, table and figure. Page numbers should be inserted once the revised manuscript is paginated in its final form.

Section/Topic	Item No.	Recommendation	Reported in the revised manuscript
**Title and abstract**			
Title and abstract	1	(a) Indicate the study's design with a commonly used term in the title or the abstract	Title ("A Cross-Sectional Analysis of the 2022–2023 KNHANES"); Abstract, Methods ("This cross-sectional secondary analysis...")
		(b) Provide in the abstract an informative and balanced summary of what was done and what was found	Structured abstract: Background/Objectives, Methods, Results, Conclusions. Revised to report the prespecified model, to state the small cell size for the one-generation other-type household estimate, to replace "social support" with "household composition", and to state that screening value requires prospective evaluation
**Introduction**			
Background /rationale	2	Explain the scientific background and rationale for the investigation being reported	Section 1. Revised so that the contribution is framed as incremental: prior Korean and KNHANES studies of overlapping middle-aged populations are acknowledged, and the contribution is located in the 2022–2023 wave, the combination of covariates, and the handling of depression

Section/Topic	Item No.	Recommendation	Reported in the revised manuscript
Objectives	3	State specific objectives, including any prespecified hypotheses	Section 1, final paragraph (aims (1) and (2))
**Methods**			
Study design	4	Present key elements of study design early in the paper	Section 2.1 (cross-sectional, descriptive correlational; secondary analysis; reported in accordance with STROBE); also stated in the Title and Abstract
Setting	5	Describe the setting, locations, and relevant dates, including periods of recruitment, exposure, follow-up, and data collection	Section 2.2 (nationwide Republic of Korea; 9th KNHANES fielded 2022–2023; raw data released January 2024); Section 2.4. Follow-up not applicable (cross-sectional)
Participants	6	(a) Give the eligibility criteria, and the sources and methods of selection of participants	Section 2.2 (adults aged 40–64 years with complete data on all analytic variables); Figure 1; Section 2.4 (stratified, multistage, probability-proportional cluster sampling)
Variables	7	Clearly define all outcomes, exposures, predictors, potential confounders, and effect modifiers. Give diagnostic criteria, if applicable	Outcome: Section 2.3.3. Predictors and covariates: Sections 2.3.1 and 2.3.2. Anxiety: GAD-7 total score of 10 or higher, described throughout as elevated anxiety symptoms and explicitly distinguished from a DSM-5 diagnosis (Section 2.3.2). Depression excluded by design and by data availability (Section 2.5)
Data sources/ measurement	8	For each variable of interest, give sources of data and details of methods of assessment (measurement). Describe comparability of assessment methods if there is more than one group	Sections 2.3.1–2.3.3 (item wording, response options, and cut-offs for every variable); Section 2.4. All variables were obtained from the same standardised KNHANES instrument for all participants
Bias	9	Describe any efforts to address potential sources of bias	Section 2.5: the covariate set is prespecified on conceptual grounds and every covariate is retained irrespective of univariate significance, replacing the significance-based rule used in the original submission; strata, clusters and pooled weights are applied. Section 2.5: the PHQ-9 is not entered, to avoid criterion contamination with the outcome

Section/Topic	Item No.	Recommendation	Reported in the revised manuscript
			and because no depression measure is common to both survey waves. Table S2 compares the prespecified and significance-based specifications; Table S3 quantifies confounding by depression; Table S4 panel A compares included and excluded participants; Section 4.1 discusses residual confounding
Study size	10	Explain how the study size was arrived at	Section 2.2 and Figure 1. No a priori sample size calculation: all KNHANES participants meeting the age criterion with complete data on every analytic variable were included (13,194 to 5153 to 4715)
Quantitative variables	11	Explain how quantitative variables were handled in the analyses. If applicable, describe which groupings were chosen and why	Sections 2.3.1 and 2.3.2. Age was grouped into 40–49 / 50–59 / 60–64 years; household income was dichotomised at the median of the quartile distribution; education was grouped into three levels; the GAD-7 was dichotomised at the validated screening cut-off of 10; physical activity was dichotomised at the WHO aerobic activity thresholds. Section 2.3.2 states that perceived stress was dichotomised at the cut point used for the KDCA's published perceived-stress indicator, and that perceived health was dichotomised at the fair/bad boundary as specified in the original analysis plan, which is not the KDCA indicator cut point; the influence of that boundary is examined in a sensitivity analysis described in Section 2.5 and reported in Table S4, panel C, and Section 4.1 notes that dichotomisation discards information
Statistical methods	12	(a) Describe all statistical methods, including those used to control for confounding	Section 2.5. Complex sample frequency analysis; Rao–Scott chi-square tests, reported for description only and not used for variable selection; complex sample multiple logistic regression with a prespecified covariate set entered simultaneously (18 parameters for 194 events)
		(b) Describe any methods used to examine subgroups and interactions	No interaction terms were fitted. Two prespecified subgroup analyses were conducted by survey wave, because a depression measure common to both waves was unavailable (Section 2.5; Table S3)
		(c) Explain how missing data were addressed	Section 2.2 and Figure 1. Complete-case analysis: 438 of 5153 age-eligible participants (8.5%) with a missing value on one or more

Section/Topic	Item No.	Recommendation	Reported in the revised manuscript
			analytic variables were excluded; variable-level counts are given in Figure 1. Section 4.1 explains why multiple imputation was not adopted as the principal analysis
		(d) If applicable, describe analytical methods taking account of sampling strategy	Section 2.5. All estimates incorporate the KNHANES stratification variable, primary sampling unit and sampling weight; for the pooled two-year analysis the annual interview and examination weight was divided by the number of pooled years, following KDCA guidance
		(e) Describe any sensitivity analyses	Section 2.5. Four prespecified sets: comparison with the significance-based specification (Table S2); wave-specific depression-adjusted models using PHQ-8 items 1–8 in 2022 and the two-week depressed-mood item in 2023 (Table S3); comparison of included and excluded participants (Table S4, panel A); three robustness analyses of the one-generation other-type household estimate (Table S4, panel B); and, added in revision, four alternative codings of perceived health (Table S4, panel C)
**Results**			
Participants	13	(a) Report numbers of individuals at each stage of study	Section 2.2; Figure 1 (13,194 surveyed; 5153 aged 40–64 years; 4715 analysed)
		(b) Give reasons for non-participation at each stage	Figure 1 (8041 outside the 40–64-year age range; 438 with a missing value on one or more analytic variables, with counts by variable). Included and excluded participants are compared in Table S4, panel A
		(c) Consider use of a flow diagram	Figure 1
Descriptive data	14	(a) Give characteristics of study participants and information on exposures and potential confounders	Sections 3.1 and 3.2; Table 1 (sociodemographic) and Table 2 (health-related and mental health)
		(b) Indicate number of participants with missing data for each variable of interest	Figure 1 (aerobic physical activity 407; economic activity 406; perceived health 394; education 99; anxiety 71; smoking 69; binge drinking 68; stress 67; suicidal ideation 67; lifetime alcohol use 66;

Section/Topic	Item No.	Recommendation	Reported in the revised manuscript
			household income 11)
Outcome data	15	Report numbers of outcome events or summary measures	Section 3.2 and Table 2: 194 participants reported suicidal ideation in the past 12 months (weighted prevalence 3.8%); 4521 did not
Main results	16	(a) Give unadjusted estimates and, if applicable, confounder-adjusted estimates and their precision. Make clear which confounders were adjusted for and why they were included	Unadjusted: Sections 3.3 and 3.4, Tables 3 and 4. Adjusted: Section 3.5 and Table 5, from the prespecified model. The covariate set and the conceptual rationale for each covariate are given in Section 2.5; no variable was included or excluded on the basis of a univariate test. Table S2 compares the prespecified and significance-based specifications
		(b) Report category boundaries when continuous variables were categorized	Sections 2.3.1 and 2.3.2 (age bands; household income quartile split; GAD-7 cut-off of 10; 150 min/week moderate or 75 min/week vigorous aerobic activity; 7 drinks for men or 5 drinks for women per occasion)
		(c) If relevant, consider translating estimates of relative risk into absolute risk for a meaningful time period	Absolute 12-month prevalence of suicidal ideation is reported for every category in Tables 3 and 4
Other analyses	17	Report other analyses done	Section 3.6 and Table S3 (wave-specific depression-adjusted models); Table S2 (specification comparison); Table S4 (comparison of included and excluded participants; alternative codings of perceived health; robustness of the one-generation other-type household estimate)
**Discussion**			
Key results	18	Summarise key results with reference to study objectives	Section 4, opening paragraphs; Section 5, first paragraph
Limitations	19	Discuss limitations of the study, taking into account sources of potential bias or imprecision. Discuss both direction and magnitude of any potential bias	Section 4.1, a dedicated Limitations subsection covering the cross-sectional design; the single-item outcome; the absence of depression adjustment and the resulting interpretation of the estimates as total associations; complete-case exclusion and possible selection bias; the small one-generation other-type household stratum; unmeasured loneliness and perceived support;

Section/Topic	Item No.	Recommendation	Reported in the revised manuscript
			dichotomisation of ordinal measures; the absence of any index of predictive performance; and restriction to community-dwelling adults
Interpretation	20	Give a cautious overall interpretation of results considering objectives, limitations, multiplicity of analyses, results from similar studies, and other relevant evidence	Section 4. A dedicated paragraph distinguishes risk-factor association from clinical risk prediction and states that discrimination, calibration and predictive value were not evaluated; the household-composition paragraph separates the observed associations from hypothesised psychosocial mechanisms; the small-stratum estimate is flagged as provisional
Generalisability	21	Discuss the generalisability (external validity) of the study results	Sections 2.2 and 2.4 (nationally representative probability sample); Section 4.1 (complete-case exclusion, and restriction to community-dwelling adults)
**Other information**			
Funding	22	Give the source of funding and the role of the funders for the present study and, if applicable, for the original study on which the present article is based	Funding statement ("This research received no external funding"). The original KNHANES is planned, funded and conducted by the Korea Disease Control and Prevention Agency (Sections 2.2 and 2.4); the Agency had no role in the design, analysis or interpretation of the present secondary analysis. The Data Availability Statement describes the KNHANES access conditions and states that the full analytic code, variable derivation rules and model specifications are provided as Supplementary File S2

Note: An Explanation and Elaboration article discusses each checklist item and gives methodological background and published examples of transparent reporting. The STROBE checklist is best used in conjunction with that article, which is freely available on the STROBE website (https://www.strobe-statement.org).

Table S2. Comparison of the prespecified and significance-based multivariable models (N = 4715)

Variables	Categories	Model A: prespecifiedOR (95% CI)	p	Model B: significance-basedOR (95% CI)	p

Variables	Categories	Model A: prespecifiedOR (95% CI)	p	Model B: significance-basedOR (95% CI)	p
Sex	Women vs men	1.13 (0.71–1.80)	0.597	not entered	—
Age group	50–59 vs 40–49	1.22 (0.80–1.86)	0.351	not entered	—
	60–64 vs 40–49	1.26 (0.73–2.15)	0.402	not entered	—
Household income	Low/lower-middle vs upper-middle/high	1.57 (1.08–2.28)	0.019	1.55 (1.08–2.23)	0.019
Education level	High school vs college or higher	1.26 (0.83–1.91)	0.270	1.29 (0.86–1.94)	0.212
	Middle school or below vs college or higher	2.13 (1.12–4.03)	0.020	2.24 (1.30–3.88)	0.004
Living arrangement	One-generation, single-person	1.56 (0.98–2.50)	0.062	1.72 (1.07–2.77)	0.026
	One-generation, couple	1.09 (0.64–1.84)	0.760	1.16 (0.70–1.93)	0.568
	One-generation, other	6.81 (3.29–14.08)	< 0.001	7.56 (3.90–14.67)	< 0.001
Marital status	Unmarried vs married	1.19 (0.62–2.29)	0.600	not entered	—
Economic activity	Inactive vs active	1.85 (1.25–2.73)	0.002	1.89 (1.27–2.82)	0.002
Lifetime alcohol use	No vs yes	0.88 (0.39–1.98)	0.761	not entered	—
Binge drinking	High vs low	1.22 (0.74–2.00)	0.440	not entered	—
Smoking status	Current vs non-current	1.52 (0.94–2.46)	0.086	1.53 (0.99–2.37)	0.054
Aerobic physical activity	Not meeting vs meeting guidelines	0.84 (0.58–1.23)	0.375	not entered	—
Perceived health status	Poor vs good	1.70 (1.13–2.55)	0.011	1.70 (1.14–2.52)	0.009
Elevated anxiety symptoms	Yes vs no	5.01 (3.02–8.32)	< 0.001	5.13 (3.11–8.47)	< 0.001
Stress perception	High vs low	8.54 (5.47–13.34)	< 0.001	8.50 (5.45–13.24)	< 0.001

Reference categories are as in Table 5. Both models were fitted on the same analytic sample (N = 4715; 194 events) under the same complex sample design. **Model A** (prespecified, reported as Table 5) retains all conceptually relevant covariates irrespective of univariate significance. **Model B** reproduces the significance-based specification used in the original submission, in which only variables significant in univariate analysis were entered; it is shown for comparison only. The two specifications agree closely on every estimate except the one-generation single-person household association, which is attenuated from 1.72 to 1.56 and crosses the conventional significance threshold under prespecified adjustment.

Table S3. Depression-adjusted sensitivity analyses

The PHQ-9 was administered only in the 2022 wave of the 9th KNHANES; the 2023 wave instead includes a single item on two-week depressed mood. Depression could therefore not be entered into the pooled model, and two parallel wave-specific sensitivity analyses were conducted. Each model contains all prespecified covariates listed in Table 5.

Panel A. 2022 subsample: adjustment for PHQ-8 (items 1–8, suicidality item omitted) — n = 2173; 92 events

Variables	Categories	Without depressionOR (95% CI)	+ PHQ-8 ≥ 10OR (95% CI)	+ PHQ-8 continuousOR (95% CI)
Sex	Women vs men	1.18 (0.59–2.36)	1.34 (0.66–2.71)	1.26 (0.61–2.61)
Age group	50–59 vs 40–49	1.23 (0.62–2.46)	1.31 (0.66–2.64)	1.39 (0.67–2.89)
	60–64 vs 40–49	1.81 (0.76–4.30)	1.88 (0.82–4.30)	2.09 (0.89–4.90)
Household income	Low/lower-middle	1.99 (1.15–3.45)	1.82 (1.02–3.24)	1.68 (0.93–3.04)
Education level	High school	1.48 (0.78–2.82)	1.53 (0.80–2.94)	1.52 (0.77–3.00)
	Middle school or below	3.00 (1.07–8.41)	3.20 (1.12–9.11)	3.00 (1.02–8.81)
Living arrangement	One-generation, single-person	1.53 (0.75–3.12)	1.39 (0.64–2.99)	1.31 (0.59–2.94)
	One-generation, couple	0.81 (0.36–1.81)	0.78 (0.36–1.71)	0.83 (0.38–1.78)
	One-generation, other	6.03 (2.09–17.37)	4.97 (1.82–13.55)	5.10 (1.87–13.95)
Marital status	Unmarried	2.14 (0.86–5.34)	2.13 (0.80–5.68)	1.95 (0.68–5.64)
Economic activity	Inactive	1.59 (0.87–2.93)	1.52 (0.84–2.76)	1.41 (0.76–2.59)
Lifetime alcohol use	No	0.32 (0.10–1.04)	0.32 (0.09–1.16)	0.37 (0.11–1.27)
Binge drinking	High	1.90 (0.89–4.06)	1.74 (0.81–3.75)	1.79 (0.83–3.88)

Variables	Categories	Without depressionOR (95% CI)	+ PHQ-8 ≥ 10OR (95% CI)	+ PHQ-8 continuousOR (95% CI)
Smoking status	Current smoker	0.85 (0.39–1.88)	0.86 (0.39–1.91)	0.87 (0.38–1.95)
Aerobic physical activity	Not meeting guidelines	0.54 (0.32–0.92)	0.56 (0.32–0.95)	0.58 (0.34–1.01)
Perceived health status	Poor	1.77 (0.97–3.23)	1.49 (0.77–2.89)	1.14 (0.57–2.28)
Elevated anxiety symptoms	Yes	**5.77 (2.61–12.75)**	**4.02 (1.62–9.94)**	**2.84 (1.10–7.37)**
Stress perception	High	**7.73 (3.87–15.44)**	**6.59 (3.31–13.12)**	**5.48 (2.64–11.37)**
Depression (PHQ-8)	≥ 10 vs < 10	—	2.92 (1.40–6.08)	—
	per 1-point increase	—	—	1.14 (1.07–1.22)

Panel B. 2023 subsample: adjustment for two-week depressed mood — n = 2542; 102 events

Variables	Categories	Without depressionOR (95% CI)	+ depressed moodOR (95% CI)
Sex	Women vs men	1.28 (0.68–2.43)	1.02 (0.54–1.94)
Age group	50–59 vs 40–49	1.15 (0.70–1.89)	1.09 (0.63–1.88)
	60–64 vs 40–49	1.00 (0.52–1.91)	0.86 (0.41–1.79)
Household income	Low/lower-middle	1.28 (0.80–2.06)	1.52 (0.90–2.59)
Education level	High school	1.02 (0.58–1.81)	0.99 (0.51–1.90)
	Middle school or below	1.64 (0.77–3.50)	1.59 (0.69–3.66)
Living arrangement	One-generation, single-person	1.48 (0.80–2.72)	0.88 (0.39–2.00)
	One-generation, couple	1.23 (0.63–2.43)	1.25 (0.63–2.47)
	One-generation, other	**7.85 (2.69–22.91)**	**7.02 (2.33–21.19)**
Marital status	Unmarried	0.63 (0.27–1.49)	0.51 (0.22–1.19)
Economic activity	Inactive	2.11 (1.28–3.50)	1.92 (1.06–3.50)
Lifetime alcohol use	No	1.68 (0.69–4.10)	1.93 (0.86–4.33)
Binge drinking	High	0.84 (0.43–1.62)	0.73 (0.35–1.54)
Smoking status	Current smoker	2.46 (1.33–4.52)	2.68 (1.44–4.99)
Aerobic physical activity	Not meeting guidelines	1.29 (0.76–2.19)	1.25 (0.70–2.22)

Variables	Categories	Without depressionOR (95% CI)	+ depressed moodOR (95% CI)
Perceived health status	Poor	1.67 (0.98–2.85)	1.32 (0.75–2.34)
Elevated anxiety symptoms	Yes	**4.96 (2.57–9.56)**	**2.37 (1.19–4.71)**
Stress perception	High	**9.09 (5.21–15.86)**	**4.32 (2.22–8.42)**
Depressed mood (≥ 2 weeks)	Yes vs no	—	14.34 (7.44–27.65)

In both waves, adjustment for depression attenuated the perceived-stress and anxiety associations by approximately 15–55%, depending on the survey wave and on whether depression was modelled dichotomously or continuously, but did not eliminate them; the one-generation other-type household association was largely unaffected; and the perceived-health and household-income associations were substantially attenuated, consistent with pathways operating through depression. In the 2022 subsample the PHQ-8 correlated with the full PHQ-9 at $r = 0.997$. Estimates in the principal analysis (Table 5) are therefore interpreted as total associations rather than as depression-adjusted direct effects.

Table S4. Robustness analyses

Panel A. Comparison of included and excluded age-eligible participants

Characteristic	Included (n = 4715)	Excluded (n = 438)	p
Sex: men / women	1934 (41.0%) / 2781 (59.0%)	228 (52.1%) / 210 (47.9%)	< 0.001
Age, mean (years)	52.7	52.3	0.228
Age group: 40–49 / 50–59 / 60–64	1693 (35.9%) / 1856 (39.4%) / 1166 (24.7%)	158 (36.1%) / 184 (42.0%) / 96 (21.9%)	0.367
Household income: low / high	1488 (31.6%) / 3227 (68.4%)	134 (31.4%) / 293 (68.6%)	0.983
Education: ≤ middle school / high school / college+	629 (13.3%) / 1841 (39.0%) / 2245 (47.6%)	43 (12.7%) / 131 (38.6%) / 165 (48.7%)	0.909
Living arrangement: single / couple / other / 2nd+ gen	571 (12.1%) / 1055 (22.4%) / 59 (1.3%) / 3030 (64.3%)	61 (13.9%) / 105 (24.0%) / 1 (0.2%) / 271 (61.9%)	0.139
Marital status: unmarried / married	377 (8.0%) / 4338 (92.0%)	29 (6.6%) / 409 (93.4%)	0.353
Binge drinking: low / high	3806 (80.7%) / 909 (19.3%)	270 (73.0%) / 100 (27.0%)	< 0.001

Characteristic	Included (n = 4715)	Excluded (n = 438)	p
Smoking: non-current / current	3889 (82.5%) / 826 (17.5%)	279 (75.6%) / 90 (24.4%)	0.001
**Suicidal ideation: yes**	**194 (4.1%)**	**13 (3.5%)**	**0.662**

Unweighted counts and column percentages; p-values from Pearson chi-square tests (Student's *t*-test for mean age). Percentages are computed among participants with the characteristic recorded. The outcome was recorded for 371 of the 438 excluded participants. Excluded participants differed on sex, smoking and binge drinking but not on age, socioeconomic position, household composition, marital status, or — critically — on the prevalence of suicidal ideation itself.

Panel B. Robustness of the one-generation other-type household estimate (n = 59; 11 events)

Analysis	Estimate for one-generation other-type household	95% CI	p
(i) Primary prespecified model (Table 5)	OR = 6.81	3.29–14.08	< 0.001
(ii) Unweighted logistic regression, profile-likelihood CI	OR = 4.85	1.88–11.71	—
(iii) Collapsed with single-person households into "one-generation non-couple household" (n = 630)	OR = 1.91	1.24–2.93	0.003

Effect of removing the category on the remaining estimates (prespecified model refitted after excluding all 59 participants; n = 4656):

Correlate	Primary model (n = 4715)	Excluding one-generation other-type (n = 4656)
High perceived stress	8.54 (5.47–13.34)	7.97 (5.11–12.45)
Elevated anxiety symptoms	5.01 (3.02–8.32)	4.95 (2.95–8.29)
Middle school education or below	2.13 (1.12–4.03)	1.93 (1.00–3.75)
Economic inactivity	1.85 (1.25–2.73)	1.89 (1.26–2.84)
Poor perceived health	1.70 (1.13–2.55)	1.71 (1.13–2.59)
Low household income	1.57 (1.08–2.28)	1.50 (1.01–2.22)
One-generation single-person household	1.56 (0.98–2.50)	1.67 (1.05–2.65)

Removing the small category leaves the remaining estimates essentially unchanged, indicating that it does not distort the model. All three approaches in the upper panel support an elevated association in this group while confirming that the design-weighted point estimate is imprecise; the finding requires replication in larger samples.

Panel C. Alternative codings of perceived health status (N = 4715)

The KNHANES records perceived health on five levels. The principal model dichotomizes it as good (very good, good, or fair) versus poor (bad or very bad). This is **not** the cut point used for the KDCA's published perceived-health indicator, which counts only "very good" and "good" as good. The model is therefore reported here under four codings of this variable.

C1. Suicidal ideation by each of the five levels

Perceived health	n	Suicidal ideation, n	Weighted %
Very good	201	4	1.31
Good	1324	21	1.42
Fair	2385	81	3.10
Bad	706	66	8.83
Very bad	99	22	22.81

Prevalence rises monotonically across the five levels, a 17-fold spread from the best to the worst category. "Very good" and "good" are indistinguishable (1.99% vs 1.59% unweighted, Fisher p = 0.561), whereas "good" and "fair" differ (1.59% vs 3.40%, Fisher p = 0.001).

C2. The prespecified model refitted under four codings of perceived health

Coding	Estimate for perceived health, OR (95% CI)	p
(i) Good + fair vs bad + very bad (principal model)	1.70 (1.13–2.55)	0.011
(ii) Very good + good vs fair + bad + very bad (KDCA indicator cut)	1.53 (0.93–2.53)	0.094
(iii) Three levels: fair vs good poor vs good	1.30 (0.77–2.19)2.07 (1.17–3.66)	0.3300.012
(iv) Five-level scale, per one-step worsening	1.42 (1.11–1.82)	0.005

C3. Effect of the coding on the other estimates

Correlate	(i) Principal	(ii) KDCA cut	(iii) Three levels	(iv) Five-level
High perceived stress	8.54 (5.47–13.34)	8.99 (5.85–13.80)	8.33 (5.37–12.94)	8.27 (5.36–12.77)

Correlate	(i) Principal	(ii) KDCA cut	(iii) Three levels	(iv) Five-level
Elevated anxiety symptoms	5.01 (3.02–8.32)	5.43 (3.28–8.98)	4.98 (3.00–8.27)	4.91 (2.95–8.17)
One-generation other-type household	6.81 (3.29–14.08)	6.79 (3.14–14.66)	6.73 (3.23–14.01)	6.70 (3.17–14.15)
Middle school education or below	2.13 (1.12–4.03)	2.15 (1.13–4.10)	2.08 (1.09–3.97)	2.02 (1.06–3.86)

Under the KDCA indicator cut (ii) the perceived-health association is attenuated to non-significance, so the significance of this particular estimate does depend on where the five-level scale is divided. After adjustment, "fair" is not distinguishable from "good" (iii), whereas the untransformed five-level scale (iv) shows a significant monotone gradient. Every other estimate in the model is essentially unchanged across all four codings. We therefore regard the perceived-health estimate as the least robust of the significant associations reported in Table 5, and the remaining findings as insensitive to this coding decision.
